# Assay Systems for Profiling Deubiquitinating Activity

**DOI:** 10.3390/ijms21165638

**Published:** 2020-08-06

**Authors:** Jinhong Cho, Jinyoung Park, Eunice EunKyeong Kim, Eun Joo Song

**Affiliations:** 1 Biomedical Research Institute, Korea Institute of Science and Technology, Seoul 02792, Korea; wlsghd1116@gmail.com (J.C.); eunice@kist.re.kr (E.E.K.); 2 Molecular Recognition Research Center, Korea Institute of Science and Technology, Seoul 02792, Korea; jypark@kist.re.kr; 3 Graduate School of Pharmaceutical Sciences and College of Pharmacy, Ewha Womans University, Seoul 03760, Korea

**Keywords:** deubiquitination assay, Ub-probes, activity-based probes, cell permeability

## Abstract

Deubiquitinating enzymes regulate various cellular processes, particularly protein degradation, localization, and protein–protein interactions. The dysregulation of deubiquitinating enzyme (DUB) activity has been linked to several diseases; however, the function of many DUBs has not been identified. Therefore, the development of methods to assess DUB activity is important to identify novel DUBs, characterize DUB selectivity, and profile dynamic DUB substrates. Here, we review various methods of evaluating DUB activity using cell lysates or purified DUBs, as well as the types of probes used in these methods. In addition, we introduce some techniques that can deliver DUB probes into the cells and cell-permeable activity-based probes to directly visualize and quantify DUB activity in live cells. This review could contribute to the development of DUB inhibitors by providing important information on the characteristics and applications of various probes used to evaluate and detect DUB activity in vitro and in vivo.

## 1. Introduction

Post-translational modifications (PTMs) contribute to the dynamic regulation of cellular processes by changing the structure and properties of proteins through the covalent modification of proteins. Ubiquitination is a versatile PTM and it is involved in various cellular processes such as protein degradation, protein–protein interaction, and cellular localization. Ubiquitination is carried out by the cascade of three enzymes, i.e., E1 (ubiquitin-activating enzyme), which mediates the activation step, E2 (ubiquitin-conjugating enzyme), which mediates the conjugation step, and E3 (ubiquitin ligase), which mediates the ligation step. These three enzymes catalyze the isopeptide bond between the Lys (K) residue on a substrate protein and the Gly residue on the C-terminus of ubiquitin, resulting in ubiquitination of a substrate [1,2]. Mono-ubiquitination affects proteins in different ways, e.g., it may regulate processes such as endocytic trafficking, inflammation, translation, and DNA repair. However, once attached to a substrate, ubiquitin can be subjected to further modifications. Ubiquitin can be ubiquitinated on seven lysine residues (K6, K11, K27, K29, K33, K48, and K63) or on the N-terminus leading to polyubiquitin chains that can encompass complex topologies. Differently linked chains have specific effects on the protein to which they are attached depending on differences in the conformations of the protein chains, which translate into different functions [3,4]. The most well-known role of ubiquitination is proteasomal degradation [4]. The K48-linked ubiquitin chain mainly serves as a protein degradation signal for the 26S proteasome, whereas other linkage-specific ubiquitin chains such as the K63-linked ubiquitin chain participate in various intracellular signal transduction pathways such as DNA damage and immune response rather than degradation. The formation of uniform ubiquitin linkages or mixed ubiquitin linkages has been reported and contributes to biological complexity [2].

Ubiquitination can be reversed via the cleavage of ubiquitin from the substrate mediated by a group of enzymes known as deubiquitinating enzymes (DUBs). Approximately 100 DUBs have been identified in the human genome and are grouped in seven different families: ubiquitin C-terminal hydrolases (UCHs), ubiquitin-specific proteases (USPs), ovarian tumor proteases (OTUs), Machado–Joseph domain proteases (MJDs), JAB1/MPN/Mov34 metalloenzymes (JAMMs, also known as JAMM/MPN+), and zinc finger with UFM1-specific peptidases (ZUFSPs), and MIU-containing novel DUB family (MINDY). Members of the JAMM/MPN + family are zinc metalloproteases, but the others are cysteine proteases also known as thiol proteases [1]. The deubiquitination process also regulates several intracellular pathways, and it is often associated with various human diseases. For example, USP2 overexpression is related to prostate cancer [5,6] and breast cancer [7]. USP7 plays multi-dimensional roles in various cancers, including prostate cancer, lung cancer, brain cancer, colon cancer, breast cancer, epithelial ovarian carcinoma, liver cancer, and leukemia [8,9,10]. Cezanne 1 is amplified in breast cancer [11]. OTUB1 promotes prostate cancer [12]. Several studies have revealed that DUBs are associated with neurodegenerative diseases. I93M and S18Y polymorphisms of UCH-L1 are associated with Parkinson’s disease (PD) [13,14]. In addition, USP9X levels are significantly lower in PD and diffuse Lewy body disease (DLBD) [15]. The dysregulation of USP14 leads to ataxia [16]. USP30 and USP35 can delay PARK2-mediated mitophagy, which leads to mitochondrial dysfunction and affects development of PD and Alzheimer’s disease [17]. Furthermore, DUBs play key roles in infectious diseases; CYLD can be targeted as an inhibitor in control of certain pathogenic inflammations [18]. USP18 overexpression restricts porcine reproductive and respiratory syndrome (PRRSV) growth [19]. The inhibition of USP14 is found to impair the replication of Dengue virus (DENV) which causes fatal Dengue hemorrhagic fever [20]. Therefore, DUBs may be considered as novel targets, and inhibitors targeting DUBs are currently being investigated by research institutes and pharmaceutical companies with some inhibitors undergoing preclinical stages. For example, ADC-01, ADC-03, HBX41108, HBX19818, P5091, and P22077, which target USP7, are under preclinical investigation as candidates for anti-cancer drugs [21]. Proteasome-associated DUBs such as PSMD14, USP14, and UCHL-5 have attracted attention as anti-cancer drug targets and several compounds, e.g., b-AP15, targeting these DUBs are in the preclinical stages of development [22,23]. The USP11 inhibitor mitoxantrone and the USP20 inhibitor GSK2643943A are in preclinical stages for development as anti-cancer drugs [22,23]. Furthermore, the USP2 inhibitor ML364 and a dual inhibitor of USP10 and USP13 (spautin 1) are in preclinical stages for development as anti-inflammatory drugs. The USP14 inhibitor IU1 and the USP30 inhibitor 15-oxospiramilactone are in preclinical stages for development as neurodegeneration-targeted drugs [21]. Considering the importance of DUBs, deubiquitination assays are vital for characterizing the ubiquitination mechanism.

To elucidate the biological roles of DUB or to evaluate the effect of DUB inhibitors, assay systems that can provide a precise assessment of the degree of ubiquitination are essential. Particularly, high-throughput screening (HTS) -based deubiquitination assay is required for more efficient screening of compounds targeting DUBs, and the global demand for new probes and methods applicable to HTS is growing as the importance of DUBs as a drug target increases. Here, we review the currently available deubiquitination assay systems. 

## 2. In vitro Deubiquitination Assays

**Figure 1:**
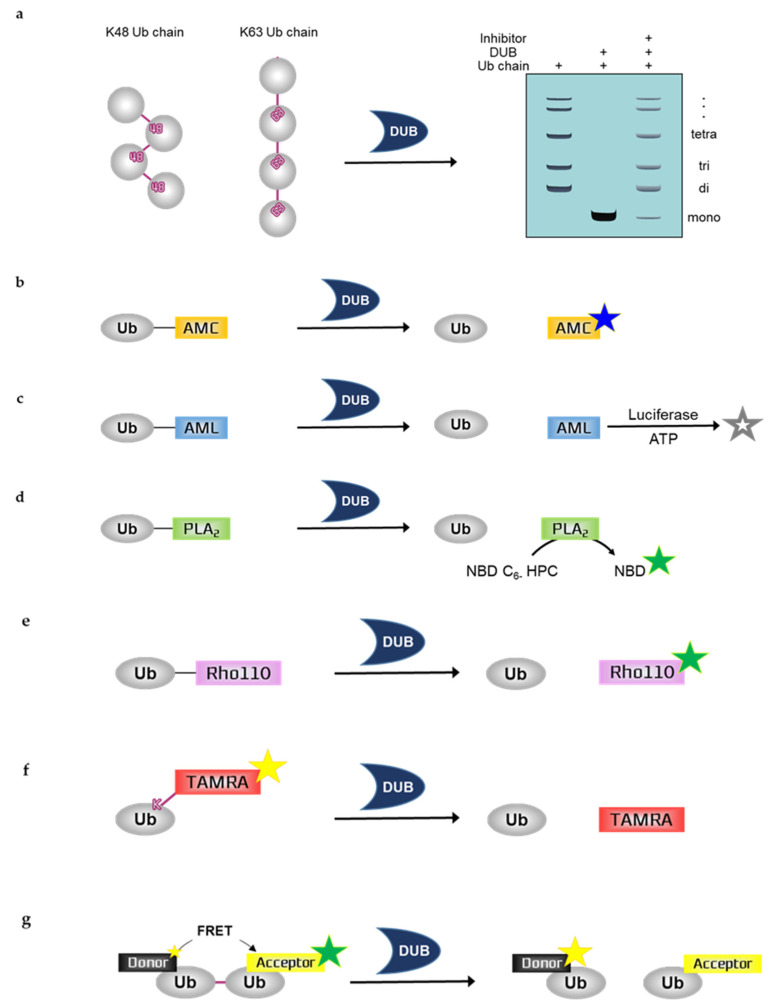
Schematic overview of in vitro deubiquitination assays. (**a**) Ubiquitin chain cleavage assay, (**b**) Ubiquitin-7-aminno-4-methylcoumarin (Ub-AMC), (**c**) Ubiquitin-aminoluciferin (Ub-AML), (**d**) Ubiquitin-phospholipase A_2_ (Ub-PLA_2_), (**e**) Ubiquitin-rhodamine110 (Ub-Rho110), (**f**) Ubiquitin-Lys-5-tetramethylrhodamine-Gly (Ub-Lys-TAMRA-Gly), (**g**) Fluorescence resonance energy transfer-based di-ubiquitin (FRET-based di-Ub). Ub; ubiquitin, DUB; deubiuquitinating enzyme, ATP; a, NBD C_6-_HPC; 2-(6-(7-nitrobenz-2-oxa-1,3-diazol-4-yl)amino)hexanoyl-1-hexadecanoyl-sn-glycero-3-phosphocholine, NBD; 7-nitrobenz-2-oxa-1,3-diazole.

Deubiquitination assay typically measures the degree of ubiquitination, thus, requiring ubiquitinated substrate and DUBs. For the assays, purified recombinant DUBs are usually used; however, immunoprecipitated DUBs from cell lysates could also be used. In a typical process of determining the activity of DUBs or DUB inhibitors, deubiquitination assays with purified recombinant DUBs are performed first, followed by deubiquitination assays with immunoprecipitated DUBs or deubiquitination assays with mammalian cells to assess and confirm intracellular activity. The use of immunoprecipitated DUBs for deubiquitination assays including ubiquitin chain cleavage assays allows the analysis of DUBs as an intracellular moiety. Recombinant proteins extracted from *Escherichia coli* are limited due to lack of PTMs such as phosphorylation [24]. However, the PTM of DUBs is very important for their activity; thus, deubiquitination assays with immunoprecipitated DUB could be performed to confirm reactivity [25,26]. In comparison with cell-based methods, in vitro deubiquitination assays produce objective and quantifiable results. Here, we describe in vitro deubiquitination assays and summarize them in Figure 1. 

### 2.1. Ubiquitin Chain Cleavage Assay

Ubiquitin chain cleavage assay can allow for the visualization of DUB activity based on the mono-ubiquitin band by SDS-PAGE including Coomassie blue staining, silver staining, or western blotting using ubiquitin antibodies. DUBs can disassemble the ubiquitin chain, forming mono-ubiquitin. Therefore, DUB activity can be measured by examining the mono-ubiquitin band on the gel. As the deubiquitinating reaction progresses, more mono-ubiquitin bands appear. To quantify the results, the band intensity of mono-ubiquitin should be analyzed by a program such as ImageJ. Ubiquitin chains in this assay are purified recombinant protein substrates and are reacted with either purified recombinant DUBs or immunoprecipitated DUBs. Ubiquitin chains can be used for various experimental purposes. These chains have different lengths depending on the types of ubiquitin, e.g., di-Ub, tetra-Ub, and hexa-Ub. Furthermore, ubiquitin chains can be classified according to the type of ubiquitin linkage. The different types of ubiquitin chains allow the analysis of ubiquitin-linkage specificity, e.g., K11, K48, or K63 linkage chains. The linkage specificity of DUBs can be determined by performing ubiquitin chain cleavage assay using different types of linkage chains. For example, MINDY-1 is a specific DUB for K48-linked ubiquitin chains [27]. USP35 mainly disassembles K11- and K63-linked ubiquitin chains and weakly disassembles K48-linked chains, indicating that USP35 has partial specificity or preference for K11- and K63-linked chains [28]. Moreover, some candidate compounds of TRABID inhibitor have demonstrated their ability in inhibiting the cleavage of hexa-K63 ubiquitin by TRABID [29].

### 2.2. Ubiquitin Probes

As mentioned above, ubiquitin substrate and DUB are needed for deubiquitination assays. In addition to ubiquitin chains, ubiquitin probes are used as ubiquitinated substrates for deubiquitination assays. Ubiquitin probes can measure and quantify the activity of DUB through various detection methods, such as fluorescence, luminescence, mass spectrometry, HPLC, SDS-PAGE, western blotting, and MALDI-TOF assay. Traditionally, the ubiquitination of target proteins has been monitored using ubiquitin that is either epitope-tagged or radiolabeled, thus requiring laborious detection methods. Currently, fluorescent or luminescent-labeled ubiquitin is frequently used for examining ubiquitination in vitro because this approach is rapid. Here, we summarize the various ubiquitin probes commonly used.

#### 2.2.1. Ubiquitin-7-Amino-4-Methylcoumarin (Ub-AMC)

Assays with Ub-AMC can visualize the DUB activity by measuring fluorescence. Ub-AMC is the most frequently used fluorescent ubiquitin substrate whose C-terminal derivative is AMC [30]. This derivative quenches the intrinsic fluorescence but releases fluorescence following hydrolysis by DUBs; fluorescence emission facilitates the real-time monitoring of the sensitivity or activity of DUBs [31]. This monitoring can be performed using 380 nm excitation and 460 nm emission wavelengths. Therefore, as the deubiquitinating reaction progresses, the fluorescence increases. However, the linkage between ubiquitin and the fluorophore AMC is not an isopeptide bond shown in the natural ubiquitin chain; and AMC is just covalently linked to ubiquitin. In a previous study, the DUB activity of USP4 was inhibited by neutral red (NR) treatment in a dose-dependent manner, which was measured by Ub-AMC analysis [32]. WP1130 inhibited purified recombinant USP5 and UCH-L1 and immunoprecipitated USP9X in a dose-dependent manner, which was also measured by Ub-AMC analysis [33].

#### 2.2.2. Ubiquitin-Aminoluciferin (Ub-AML)

Assays with Ub-AML can visualize DUB activity by measuring the luminescence. Ub-AML is a ubiquitin reporter substrate whose C-terminal derivative is AML. Luciferin is released from Ub-AML via DUB activity. Subsequently, ATP and luciferase are added to produce a luminescent signal in proportion to DUB activity [34]. Therefore, as the deubiquitinating reaction progresses, the luminescence increases. Luminescence assays are strong and sensitive; thus, these assays are beneficial against noise contamination compared with traditional fluorophore-based assays with low sensitivity. However, the linkage between ubiquitin and the luminophore AML is not an isopeptide bond shown in the natural ubiquitin chain; and AML is just covalently linked to ubiquitin. Ub-AML has shown enhanced sensitivity in detecting DUBs that were otherwise difficult to detect by Ub-AMC [34]. Moreover, a benzophenazine compound, OR141, inhibited the cleavage of Ub-AML by DUBs present in purified 19S proteasome [35].

#### 2.2.3. Ubiquitin-Phospholipase A_2_ (Ub-PLA_2_)

Assays with Ub-PLA_2_ can visualize the DUB activity by measuring fluorescence. Ub-PLA_2_ is a ubiquitin reporter substrate whose C-terminal is fused to PLA_2_. PLA_2_ requires a free amino terminus to be catalytically active. Active PLA_2_ cleaves the 2-acyl linkage of 3-sn-phosphoglycerides in a Ca^2+^-dependent reaction and is dose-dependently detected by the release of the fluorophore 7-nitrobenz-2-oxa-1,3-diazole (NBD) from 2-(6-(7-nitrobenz-2-oxa-1,3-diazol-4-yl)amino)hexanoyl-1-hexadecanoyl-sn-glycero-3-phosphocholine (NBD C_6-_HPC) [36]. Therefore, DUBs can produce a fluorescent signal by cleaving the ubiquitin C terminus of Ub-PLA_2_ to give a free amino terminus for catalytically active PLA_2_. Reaction monitoring can be performed using 460 nm excitation and 534 nm emission wavelengths. In a previous study, the nonselective isopeptidase inhibitor NSC 632839 did not inhibit the PLA_2_ reporter enzyme, indicating that the reported inhibition was selective for isopeptidases but not for the PLA_2_ reporter reaction [36]. However, the linkage between ubiquitin and the fluorophore PLA_2_ is not an isopeptide bond shown in the natural ubiquitin chain; and PLA_2_ is just covalently linked to ubiquitin. A deubiquitination assay with Ub-PLA_2_ demonstrated that the DUB inhibitor P22077 inhibited USP7 and USP47, which have high similarity. In contrast, another DUB inhibitor, PR-619, exhibited a limited inhibitory effect on multiple DUBs [37].

#### 2.2.4. Ubiquitin-Rhodamine110 (Ub-Rho110)

Assays with Ub-Rho110 can visualize DUB activity by measuring fluorescence. Ub-Rho110 is a ubiquitin substrate whose C-terminal derivatives are Rho110. While rhodamine is in the Ub-Rho110 moiety, it is di-substituted thereby quenching the intrinsic fluorescence. However, mono-substituted rhodamine, which exhibits intense fluorescence, is released by the DUBs, thus allowing the real-time monitoring of the sensitivity or activity of DUBs. This monitoring can be performed using 485 nm excitation and 535 nm emission wavelengths [38]. With these longer wavelengths, the risks of artifacts in auto-fluorescence are reduced. However, the linkage between ubiquitin and the fluorophore Rho110 is not an isopeptide bond shown in the natural ubiquitin chain; and Rho110 is just covalently linked to ubiquitin. The USP7-selective inhibitory effects of XL188 on 41 purified DUBs were measured by Ub-Rho110 [39]. In another study, Ub-Rho110 confirmed that USP14 aptamers specifically inhibited the DUB activity of USP14; however, they failed to inhibit the DUB activities of UCHL3, USP47, USP5, and UCHL5/UCH37 [40].

#### 2.2.5. Fluorescence Polarization (FP)-Based Ubiquitin-Lys-5-Tetramethylrhodamine-Gly (Ub-Lys-TAMRA-Gly)

FP is a technique based on the difference between the polarized fluorescent light emitted when a fluorescent-labeled molecule is highly excited by polarized light and the less polarized fluorescent light emitted when a fluorescent-labeled molecule is cleaved by DUBs. Therefore, the signal is inversely proportional to DUB activity. The higher the DUB activity, the lower the emitted fluorescence. FP assays are readily adaptable to HTS, which would be useful for the screening of DUB inhibitors [41,42,43]. 

Assays with Ub-Lys-TAMRA-Gly can visualize DUB activity by measuring fluorescence. TAMRA is a fluorophore, and Ub-TAMRA is a ubiquitin substrate whose derivative is TAMRA. The Lys-Gly sequence is modified by TAMRA and is linked to ubiquitin through an isopeptide bond with a lysine side-chain, thus mimicking ubiquitinated substrates. It is useful for studying C-terminal ubiquitin hydrolytic activity over a continuous period at longer wavelengths. Ub-Lys-TAMRA-Gly has been prepared by chemical synthesis, and this probe allows the detection of ubiquitination based on fluorescence [44]. The activity of TAMRA-labeled ubiquitin is validated by the cleavage of the fluorophore TAMRA from ubiquitin after incubation with DUBs. The high molecular weight TAMRA-labeled ubiquitin emits highly polarized light when it is excited. After cleavage by DUBs, the fluorophore, which is covalently attached to ubiquitin, emits less polarized light. Thus, higher DUB activity is associated with reduced fluorescence. DUB activity is monitored based on these changes in polarization using 544 nm excitation and 572 nm emission wavelengths. The DUB activities of USP7, UCH-L3, USP21, and OTU were measured by deubiquitination assays with Ub-Lys-TAMRA-Gly [44]. The DUB activities of USP2, vOTU, OTUD3, OTUB2, and OTUD6A were also measured by this assay [45]. If the N-terminal TAMRA-tagged ubiquitin is transfected to cells by electroporation for cell-based assays, it can be detected by microscopy or SDS-PAGE [46].

#### 2.2.6. Fluorescence Resonance Energy Transfer (FRET)-Based Di-Ub Probes

Similar to ubiquitin chain cleavage assays, deubiquitination assays with di-Ub probe allow a better understanding of the DUB linkage-type preference [47]. The ubiquitin chain linkage specificity of a DUB determines its function in signaling mechanisms, and di-Ub probes can be labeled with a fluorescent molecule for the absolute quantification of the chain cleavage specificity of the DUB. Fluorescent labeling is difficult with polyubiquitin chains; thus, di-Ub is usually used for the quantification of the linkage specificity of a DUB. Fluorescent TAMRA, AMC, and Rho110 can be labeled with di-Ub. The FRET-based di-Ub deubiquitination assay also involves the use of fluorescent-labeled di-Ub probes. For example, the deubiquitinase OTUD2 showed specificity for K11-linked di-Ub, which is labeled with either TAMRA or AMC [48]. FRET is a measuring tool for observing two fluorophores of different colors. Energy transfer occurs over distances less than 10 nm and allows the detection of protein–protein interactions and protein conformational changes [49]. FRET-based di-Ub comprises two ubiquitin modules, one equipped with a donor fluorophore and the other as an acceptor. Intrinsic fluorescence from the donor fluorophore is transferred to the acceptor fluorophore thereby emitting fluorescence. These two ubiquitin modules are specifically linked to one of the seven lysine residues by an isopeptide bond. In the presence of a DUB, the FRET-based di-Ub pair is cleaved, resulting in the loss of the FRET signal and the consequent decrease in acceptor emission [50,51]. Therefore, as the deubiquitinating reaction progresses, the fluorescence emission of the acceptor and donor is decreased and increased, respectively. FRET pairs of 5-carboxyrhodamine110 (Rho110) as the donor and 5-carboxytetramethylrhodamine (TAMRA) as the acceptor showed that loss of the FRET signal was caused by TRABID-mediated di-Ub cleavage [50]. In addition, FRET-based nanoparticles were used for monitoring DUB activity. The UCH-L1 inhibitor IS-1 (an isatin O-acyl oxime compound) was observed to inhibit the DUB activity of UCH-L1 towards mesoporous silica nanoparticle-terbium-UbR (MSN-Tb-UbR), which was prepared by modifying rhodamine B-labeled Ubs (Ub-Rs) (the acceptor) on the surface of MSNs-loaded with Tb^3+^-complexes (the donor). This modified assay showed good sensitivity and selectivity for monitoring the effect of the UCH-L1 inhibitor [52]. 

#### 2.2.7. Limitations

Fluorescence-based deubiquitination assays have been found to have a few limitations. The most common and widely used Ub-AMC has a relatively narrow wavelength range compared to other Ub-based probes, and the excitation wavelength is in the UV range. Therefore, it is not suitable for drug discovery because it can excite many screening compounds and cause false positives [53]. Unfortunately, it cannot be efficiently hydrolyzed by the USP enzymes, the largest group of DUBs [54]. Ub-Rho110, which exhibits a red-shifted fluorescence in both the excitation and emission spectra would be an excellent alternative to Ub-AMC because it provides a considerable improvement in detection. Analysis with Ub-AMC or Ub-Rho110 is not suitable for experiments that require high concentrations of enzymes and/or low concentrations of substrates, such as optimization of lead compounds [34]. The FRET assay developed for HTS is not only difficult to apply to a multi-well plate format where the end point can be read directly, but also requires special reagents and equipment. In addition, there is a disadvantage that signal loss may occur during the reaction [54]. The wavelength range of the Ub-PLA_2_ assay is better suited for drug discovery than that of the Ub-AMC assay. However, in an experiment screening commercial libraries for USP7 inhibitors using this assay, it could not be distinguished whether the inhibitors found inhibited USP7 or PLA_2_, which limits its broader applicability [55]. Unlike other assays, Ub-AML assay requires the external addition of luciferase [47].

## 3. Deubiquitination Assays for Mammalian Cells

**Figure 2:**
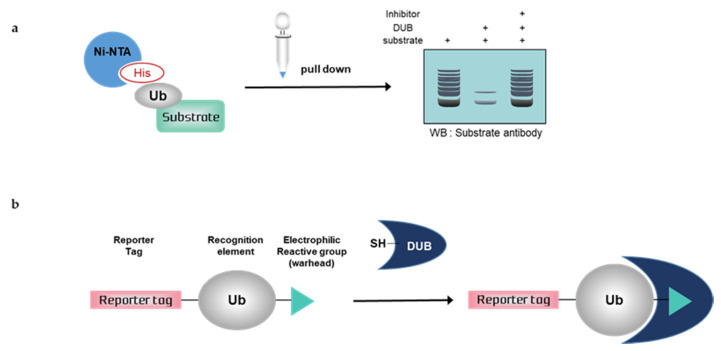
Schematic overview of deubiquitination assays with mammalian cells. (**a**) Nickel pull-down assay, (**b**) Activity-based probes (ABPs). Ub; ubiquitin, His; histidine, DUB; deubiquitinating enzymes, SH; sulfhydryl group

Deubiquitination assays with mammalian cells are performed using mammalian cell lysates or live cells. This allows the monitoring of DUB activity within the intracellular environment. In cell lysates, DUB retains its native form, either bound to interacting proteins required for proper folding or multimeric assembly, or modified by “authentic” human-like PTMs such as phosphorylation [56]. Therefore, deubiquitination assays with mammalian cells can confirm their native activity within the entire intracellular environment. Furthermore, the ubiquitin chain cleavage assay can be performed with immunoprecipitated DUBs, which would reflect their activity within the intracellular environment. For DUB studies, it is recommended to validate the activity of the DUB or the DUB inhibitor by deubiquitination assays with mammalian cells to assess their actual activity within the intracellular environment. Nickel pull-down assays are typical deubiquitination assays with mammalian cells; however, the use of activity-based probes (ABPs) is also increasing. Here, we review deubiquitination assays with mammalian cells for assessing the intracellular activity of DUBs or the effect of DUB inhibitors (Figure 2). 

### 3.1. Nickel Pull-Down Assays

Nickel pull-down assays can visualize the ubiquitination of the substrate protein by western blotting using anti-substrate antibodies. In these assays, the substrate, DUB, and His-tagged ubiquitin are overexpressed in cells, and ubiquitinated cellular proteins are extracted by Ni-NTA agarose and analyzed by western blotting. Ni-NTA agarose is a nickel-charged affinity resin for purifying proteins tagged with a polyhistidine (6 × His) sequence. His residues of the His-tagged protein bind to the immobilized nickel ions with high specificity and affinity [57], and other proteins pass through the matrix. A ubiquitinated substrate can generate high-molecular weight bands. Consequently, quantification is usually difficult, as is a comparison with another sample. If the western blot signal is too weak, autoclaving the transferred membrane before blocking could improve the signal of the ubiquitinated substrate during western blotting. Furthermore, the ubiquitination of substrates and deubiquitinating activity of DUBs could be confirmed using site mutants of ubiquitin^K^, e.g., only the Lys48 residue is replaced with Arg and all Lys residues are replaced with Arg except Lys48 [58]. Nickel pull-down assays demonstrate ubiquitinating or deubiquitinating activity within the intracellular environment. Therefore, the assay allows us to examine the effect of DUB depletion on substrate ubiquitination. In the case of DUB depletion, the decreased ubiquitination of a substrate would be attenuated. This confirms the intracellular deubiquitinating activity of DUBs. However nickel pull-down assays are limited as they can only be used for known substrates and thus are not suitable for HTS. The Ni-NTA pull-down assay was used to measure the activity of USP47 towards its substrate, RPS2 [59]. In another study, the assay showed that HBX 19818 inhibited USP7, and the ubiquitination of MDM2, a substrate of USP7 was increased by HBX 19818 treatment in a dose-dependent manner [60]. 

### 3.2. Activity-Based Probes (ABPs)

Deubiquitination assays with mammalian cells and ABPs can confirm DUB activity by SDS-PAGE using anti-DUB antibodies or antibodies to tag the probes. Activity-based DUB probes (DUB ABPs) include various electrophiles, substrates, and N-terminal tags. Propargylamide (PA or Prg), vinylmethyl ester (VME), and vinylsulfone (VS) are typical electrophiles and their classification is summarized in Table 1.

When ABPs are incubated with mammalian cell lysates, they form a covalent bond with the active site of DUBs. Monoubiquitin probes are widely used; thus, a DUB bound to the probes is shifted to a higher molecular weight position on the gel. This upper band suggests that the active DUB is well labeled with the probes. As the extent of probe labeling depends on the activity of the DUB, the more intense the probe-bound band, the higher the DUB activity. When the DUB is inhibited by a DUB inhibitor, the upper band disappears or shows a lower band intensity [62,63]. In contrast to Ni-NTA assays, ABP-based assays are performed with untransfected whole-cell lysates; thus, HTS is possible.

### 3.3. Cell-Permeable ABPs

A number of probe designs have been reported since the development of the first DUB-related ABP; however, none of them showed cell permeability, which may be attributed to the large size of the recognition element in the probes [61]. ABPs for most proteases require only a short peptide recognition element; however, DUB ABPs require a full-length ubiquitin [61,64]. Hence, to date, DUB ABPs have only been used with cell lysates. However, cell lysis causes the large-scale dilution of the cytosols, which in turn can lead to the dissociation of protein complexes with the loss of activity [65]. Therefore, DUBs need to be labeled in their native cellular environment, where their intracellular location, protein–protein interactions, and activity can be maintained. To fully understand the function of DUBs in a physiological environment, cell permeability issues should be addressed.

#### 3.3.1. Delivery of ABPs to Cells

Previously, pore-forming toxin and electroporation were used to deliver DUB ABPs that could not pass through the cell membrane into cells. Claessen et al. were the first to use perfringolysin O (PFO), a pore-forming toxin that binds to cholesterol in the plasma membrane and forms a large pore complex, to deliver their developed catch-and-release ubiquitin ABP, which is a Ub-VME derivative with a cleavable linker (release motif) attached to a biotin affinity handle (catch part), into cells [65]. When the cells are exposed to mild hypotonic conditions and incubated with PFO and the catch-and-release ubiquitin ABP, the probe is transferred into the cells through the pores [65,66]. Electroporation has been used to directly monitor the cascade-dependent enzymatic activity of Ub-conjugating enzymes in cells. Imaging-based experiments using the Cy5-UbDha probe could allow the measurement of ubiquitination in cells [67]. However, these methods may affect ubiquitination and the signaling cascade, thus disrupting cell homeostasis [68].

#### 3.3.2. Cell-Penetrating Peptide (CPP)-Based ABPs

Recently, ABPs that can cross the cell membrane have been developed. Attaching a CPP to DUB ABPs allows the probes to pass through the cell membrane. CPPs have been reported as versatile delivery vehicles for cell-impermeable molecules [69]. Gui et al. synthesized a novel DUB ABP containing a disulfide-linked cyclic polyarginine (cR10) peptide that is known to enhance the cellular uptake of proteins at the N-terminus of the probe [69,70]. CPP-based DUB ABPs have been used for proteome-wide DUB profiling in combination with quantitative mass spectrometry in a previous study. The study confirmed 10 DUBs in cell lysates using CPP-based DUB ABPs; a total of 27 DUBs were identified in the intracellular DUB profiling experiment, including the 10 DUBs found in cell lysates. Safa et al. generated another CPP-based DUB ABP by conjugating a β-hairpin sequence motif (RWVRVpGRWIRQ) to a DUB recognition element consisting of the last four amino acid residues (LRGG) of ubiquitin [71,72]. The β-hairpin motif provides enhanced protease resilience, thus allowing the dynamic measurements of the activity of a DUB over a long period of time in its intact form. Taken together, CPP-based DUB ABPs could help identify the biological function and activity of DUBs in a physiological environment, and they are suitable for HTS and the discovery of DUB inhibitors [70].

#### 3.3.3. Small Molecule-Based ABPs

As mentioned above, the requirement of a full-length ubiquitin as a recognition element is the main reason why DUB ABPs cannot pass through cell membrane. To address this problem, the use of small molecule-based DUB ABPs has been considered. The idea of using small molecule-based DUB ABPs was proposed during the development of DUB inhibitors. Small molecules covalently bound to DUBs have been modified by the addition of a reporter group e.g., fluorescent molecules and used for competitive activity-based protein profiling (ABPP).

A small molecule-based DUB ABP, based on a chloroacetylpyrrole scaffold, was first introduced by Ward et al., which had an inhibitory effect on USP4 and USP11 [62]. This inhibitory compound was used as a DUB ABP for quantitative target engagement in live cells with ABPP methods, and it labeled a total of 12 DUBs in the cells. 

Small-molecule covalent inhibitors of ubiquitin carboxy-terminal hydrolases (UCHs), another DUB subfamily, are useful as novel cell-permeable ABPs for identifying and quantifying target protein in cells. Panyain et al. developed a cyanamide-containing inhibitor for UCHL1. This inhibitor and its alkyne-tagged analog, IMP1720, labeled the catalytic Cys residue of UCHL1 in an activity-dependent manner and provided a new tool to examine UCHL1 activity in several cell types [73]. Another small molecule inhibitor for UCHL1 has been reported by Geurink et al. They synthesized several compounds including 6RK73, 8RK54, and 8RK64 with a cyanamide moiety based on a collection of cyanopyrrolidine-based inhibitors for UCHL1. These compounds selectively inhibited UCHL1 in the presence of other DUBs in live cells [74]. 

There have been limited reports of effective small-molecule DUB ABPs. Nevertheless, the development of small-molecule DUB ABPs is regarded as vital for the application of DUB ABPs at low concentrations (nanomolar levels) in cells. However, strong off-target effects and toxicity may limit the utility of these probes in living systems. If these problems are addressed, small-molecule-based ABPs will be a potent cell-permeable tool with selective DUB activity for target identification in cells.

### 3.4. Limitations

All DUBs bind to ubiquitin adjacent to their catalytic site. Therefore, ABPs for DUBs typically include full-length ubiquitin as a substrate with a Cys reactive electrophilic group. However, some reports revealed that DUB ABPs also label various non-DUB proteins [75,76,77]. It is unclear whether these proteins covalently bind to the probes or interact through non-covalent interactions with the probes or other probe-labeled DUBs. Furthermore, Wang et al. reported that OTUB1 reacted with Ub-VS on a non-catalytic Cys residue, suggesting that DUB ABPs may not always react with DUBs at their active site [78]. In addition, the ubiquitin chain linkage specificity of DUBs may be determined by the development of di-Ub ABPs [79]. However, despite various efforts, ABPs are still limited in the analysis of DUB specificity towards target proteins and the selectivity between DUBs [80,81,82].

The use of CPPs with ABPs to pass through the cell membrane has also been reported to substantially disrupt cell membrane integrity, leading to the formation of non-physiological subcellular compartments [83]. Indeed, unexpected pore formation by CPPs can disrupt cell homeostasis, inducing cell death processes such as apoptosis and necrosis [84]. In addition, the CPP-based probe method requires a large amount of probes (>10 nM); however, analysis of the cellular target engagement and selectivity of novel DUB inhibitors require the ABP labeling of a wide range of DUBs at low concentration [69].

Small-molecule-based ABPs have strong off-target effects, affecting proteins such as non-DUB proteins, which could induce cellular toxicity. For example, b-AP15, a known inhibitor of USP14 and UCH-37 [85], and its analog, VLX1570, could react with multiple proteins in a non-specific manner, causing intracellular toxicity [86]. Through both a quantitative proteomic approach and immunoblot assay, Ward et al. found that these inhibitors could target various proteins, resulting in the formation of complexes with a higher molecular weight. Particularly, VLX1570 was strongly covalently linked to CIAPIN1. VLX1570 formed a complex with CIAPIN1, leading to the aggregation of CIAPIN1 in intact cells [86]. As CIAPIN1 exerts anti-apoptotic effects [87], its aggregation may induce cellular toxicity. In addition, the minor off-targets (UCHL3 and FGFR2) of IMP1720 were identified through proteome-wide competitive ABPP [73]. Geurink et al. reported that their UCHL1 inhibitors reacted with the non-DUB protein DJ-1 (PARK7), a major off-target [74].

## 4. Conclusions

The profiling of DUB activity using various probes is important for characterizing novel DUBs and identifying DUB inhibitors. Since each probe has different reactivities and specific applications, it is necessary to understand their features and efficacy. We have summarized deubiquitination assays according to the detection methods (Table 2) and purpose (Table 3). Thus far, most DUB assays have been performed using cell lysates or purified proteins. Since this is greatly different from the native cell environment, there are limitations in determining DUB activity and function. A method capable of measuring DUB activity in living cells is essential. The development of new tools such as cell-permeable DUB probes will allow us to explore a wide range of DUB processes. In addition to the accurate measurement of DUB activity by these assays, insights into the biological role of DUBs based on knockdown experiments, PTMs, and catalytic mutants [25,26,59,88] are needed to determine the feasibility of DUB as a target for new drugs.

## Figures and Tables

**Table 1:** Classification of ABPs [61].

Reporter Tag		Cys-Reactive Electrophilic Warhead		
Affinity	Fluorescent	Direct Addition	Conjugate Addition	Nucleophilic Substitution
HA	Cy5	Nitrile (Ub-CN)	Vinyl methyl ester(Ub-VME)	Chloroethylamine (Ub-Cl)
Biotin	TAMRA	Propargyl amide (Ub-PA/Prg)	Vinyl methyl sulfone (Ub-VS)	Bromoethylamine (Ub-Br2)
Flag	TER	Aldehyde (Ub-al)	Vinyl cyanide (Ub-CN)	Bromopropylamine (Ub-Br3)
-	-	-	Dehydroalanine (Ub-Dha)	Acyloxymethyl ketone (Ub-TF_3_BOK)
-	-	-	-	α-Amino-β-lactone (Ub-Lac)

**Table 2:** Deubiquitination assays according to detection methods.

Detection Method		Ubiquitin Substrates	Ref.
in vitro deubiquitinating assay	Fluorescence	Ub-AMC	[30,31,32,33,89]
		Ub-PLA_2_	[36,37]
		Ub-Rho110	[38,39,40,90]
		FP-based Ub-Lys-TAMRA-Gly	[44,45,46,53]
		FRET di-Ub	[50,51,52]
	Luminescence	Ub-AML	[34,35]
	Gel staining	Ubiquitin chain	[27,32,77]
Deubiquitinating assay with mammalian cells	Western blotting	Ubiquitin chain (with immunoprecipitated DUB)	[28,29,91]
		Nickel pull-down assay	[59,60,92]
		Activity-based probes	[37,62,93]
	Mass spectrometry	Activity-based probes	[37,74,94]
	Microscopy (fluorescence)	Activity-based probes (cell permeable only)	[70,95,96]

**Table 3:** Deubiquitination assays according to experimental purposes.

Purpose	Deubiquitination Assays
Substrate specificity	Nickel pulldown assay
Ubiquitin chain linkage specificity	Ubiquitin chain cleavage assay, Nickel pulldown assay, FRET di-Ub
Kinetics	Ub-AMC, Ub-AML, Ub-PLA_2_, Ub-Rho110, FP-based Ub-Lys-TAMRA-Gly, FRET di-Ub
DUB activity	Ubiquitin chain cleavage assay, Nickel pulldown assay, ABP

## Abbreviations

DUB	Deubiquitinating Enzyme
ABP	Activity-Based Probe
HTS	High-Throughput Screening
Ub	Ubiquitin
AMC	7-amino-4-methylcoumarin
AML	Aminoluciferin
PLA_2_	Phospholipase A_2_
NBD	7-nitrobenz-2-oxa-1,3-diazole
NBD C_6-_HPC	2-(6-(7-nitrobenz-2-oxa-1,3-diazol-4-yl)amino)hexanoyl-1-hexadecanoyl-sn-glycero-3-phosphocholine
Rho	Rhodamine 110
FP	Fluorescence Polarization
TAMRA	5-tetramethylrhodamine
FRET	Fluorescence Resonance Energy Transfer
PA	Propargylamide
VME	Vinylmethyl Ester
VS	Vinylsulfone
PFO	Perfringolysin O
CPP	Cell-Penetrating Peptide
